# The Effect of Longan Peel and Seed on Wheat Starch and the Quality of Longan Cake

**DOI:** 10.3390/polym17162259

**Published:** 2025-08-21

**Authors:** Yi-Shan Chen, Yang Xiao, Heng-Yu Liang, Nan Chen, Hao-Xiang Gao, Wei-Cai Zeng

**Affiliations:** 1 Antioxidant Polyphenols Team, Department of Food Engineering, Sichuan University, Chengdu 610065, China; 2 The Key Laboratory of Food Science and Technology of Sichuan Province of Education, Sichuan University, Chengdu 610065, China

**Keywords:** Longan, by-products, quality, wheat starch, physicochemical properties

## Abstract

In the present study, the effects of longan peel and seed on the quality of longan cake were determined, and the effects of longan peel extract (LPE) and longan seed extract (LSE) on the physicochemical properties of wheat starch were also measured. Furthermore, the phenolic profile and antioxidant activities of these extracts were observed. The results showed that both longan peel and seed could improve the color, texture, and volatile flavor compounds of longan cake. In addition, the properties of wheat starch, including gelatinization characteristics, thermogravimetric analysis, rheological properties, solubility, swelling power, water/oil-holding capacity and iodine binding ability, were all affected by LPE and LSE significantly. Both LPE and LSE had high contents of total phenols (LPE: 71.05 ± 2.57 mg/g, LSE: 78.49 ± 5.21 mg/g) and total flavonoids (LPE: 286.27 ± 6.04 mg/g, LSE: 423.21 ± 7.69 mg/g). Gallic acid, ellagic acid, and ellagic acid 4-O-α-l-arabinofuranoside were identified as the main phenolic compounds of LPE, while those of LSE were gallic acid, ellagic acid, ellagic acid 4-O-α-l-arabinofuranoside and (-)-epicatechin. Furthermore, LPE and LSE both exhibited good antioxidant activities to scavenge free radicals and showed high reducing power. All results suggest that longan peel and seed are rich in phenols and can improve the properties of starch so as to enhance the quality of starch product, which shows their potential application in food and chemical industries.

## 1. Introduction

Bakery food is becoming more and more popular around the world due to its good taste and special flavor. Wheat flour is the main ingredient of bakery food and has a high starch content [1]. In order to improve the quality and sensory characteristics [2,3,4] of bakery food, some additives are employed. It has been reported that synthetic agents are effective in improving the quality of bakery food, such as sodium stearoyl lactylate and azodicarbonamide, which can improve the baking properties of bread [5,6]. However, the potential toxicity of synthetic agents in the human body is being considered and is a cause for worry. Therefore, safe compounds from natural sources such as vegetables and fruits [7,8] have garnered significant interest owing to their effective capability and various functions (such as antioxidant activity [9,10], anti-inflammatory properties [11] and metabolic disease prevention [12]), which may be an alternative approach.

Longan cake, a conventional Chinese bakery product, attracts significant consumer preference for its nutritional density and cultural symbolism. Longan pulp and wheat starch are the main raw materials for its preparation. Thus, during its production, longan peel and seed are generated and discarded as by-products. With the increasing production of longan cake, more and more longan peel and seed are generated, and their valuable utilization is beginning to attract attention. It has been reported that both longan peel and seed are rich in phenolic compounds [13,14] and exhibit various functions for human health [15,16], including antioxidant, antihypertensive, and antihyperlipidemia effects. Phenolic compounds have been further demonstrated to enhance the quality of starch products via molecular interactions [17,18,19]. Thus, there is a hypothesis that longan peel and seed can be employed as natural agents to improve the quality of longan cake during its processing and storage. This approach is beneficial for the resource utilization of processing waste while improving product quality [20,21]. Therefore, as a series of studies about the valuable applications of by-products in longan production, the present study was made to determine the effects of longan peel and seed on the quality of longan cake, and the potential action mechanism involved was also observed.

In order to adhere to the aims of the present study, longan peel and seed were employed as materials to prepare longan cake, and their effects on the color, texture, and volatile flavor profiles of longan cake were determined. In addition, their effects on the physicochemical properties of wheat starch were measured. Furthermore, the phenolic profiles and antioxidant activities of extracts from longan peel and seed were evaluated to explore the potential action mechanism. The present study wants to provide some beneficial results and scientific support for the valuable applications of longan in food and relevant industries, and it also provides a theoretical basis for using longan by-products to design novel functional foods.

## 2. Materials and Methods

### 2.1. Materials and Reagents

Fresh longan was collected in July 2023 from Luzhou, China. Wheat starch was purchased from a local supermarket in Chengdu, China. 2,2-azino-bis(3-ethylbenzathiazoline-6)-sulphonic acid (ABTS) and 2,2-diphe -nyl-1-picrylhydrazyl radicals (DPPH) were purchased from OriLeaf (Shanghai, China). The water used was purified by a UPT-II-5T water purifier (ULUPURE, Chengdu, China). All other chemical reagents were analytical grade.

### 2.2. Preparation of Longan Cake with Longan Peel and Seed

#### 2.2.1. Preparation of Longan Peel Powder and Seed Powder

Longan peel, seed, and pulp were manually separated and collected from fresh longan fruits. The peel and seed were washed with water and then crushed into powder. After that, the powders were all air-dried (25 °C, 7 d) for further tests.

#### 2.2.2. Preparation of Longan Cake

Butter (54 g) and shortening (36 g) were mixed in a SM-101 blender (Jiuyang Co., Ltd., Hangzhou, China) for 3 min. Then, egg (20 g) was incorporated and the mixture was creamed together for 3 min. Low-gluten flour (90 g), skim milk powder (20 g), and a certain amount of longan peel powder or seed powder (0, 50, 100, 150, and 200 g/kg pastry dough) were added into the mixture and then homogenized using a JJ-1 homogenizer (Weiss, Suzhou, China) at a speed of 600 r/min for 5 min to form a dough.

Longan pulp was mechanically homogenized into a slurry with an MDG-35LAA pulper (Jiuyang Co., Ltd., Hangzhou, China) for 5 min. The slurry was continuously heated at 100 °C in an HH-6S water bath (yilinkexue, Shanghai, China) for 30 min until a viscous, paste-like consistency was attained.

We rolled the filling (7 g) into a round shape and wrapped it with an equal mass of pastry crust to form raw dough. After that, the raw dough was rounded again and then placed into a mold (30 mm × 30 mm × 30 mm). These unbaked products were transferred to the oven and baked at 180 °C for 15 min. After baking, all longan cake samples were naturally cooled to 25 °C indoors for further tests.

### 2.3. Determination of Texture Properties and Color of Longan Cake

The sample was cut into a 20 mm × 20 mm × 20 mm cube, and the texture characteristics of the sample were determined by puncture experiments with a TA-XT2 express texture analyzer (BosinTech, Shanghai, China). A P/2N probe was used to measure the texture of longan cake (including the hardness of the pastry and filling and the adhesiveness of the filling), with a pre-test rate of 1 mm/s, an in-test rate of 2 mm/s, and a post-test rate of 10 mm/s. The probe displacement was 10 mm, the puncture volume was 50%, and the trigger force was 5 g. All tests were measured at 25 °C. Three samples were measured for each indicator, and five identical points were selected for each sample.

The color of the surface of longan cake was measured using a DS-499 color difference meter (Caipu Technology Co., Ltd., Shanghai, China) with a D65 light source and the results were recorded using L* (lightness), a* (red–green value), and b* (yellow–blue value). The *ΔE* (color difference) values were calculated as *ΔE* = [(L* − *L*_0_)^2^ + (a* − *a*_0_)^2^ + (b* − *b*_0_)^2^]^1/2^, where *L*_0_*, a*_0_ and *b*_0_ were the color of the control, and L*, a*, and b* were the color of the sample. All tests were measured on the same part of the sample, and each sample was tested five times.

### 2.4. Determination of Volatile Flavor Components of Longan Cake

The volatile flavor components of longan cake were analyzed using headspace solid-phase microextraction coupled with gas chromatography–mass spectrometry (HS-SPME-GC-MS). Briefly, the sample (2 g) was placed in a headspace (20 mL) vial and equilibrated at 45 °C in a water bath for 15 min. The 57550-U SPME fiber (Merck KGaA, Darmstadt, Germany) was then exposed to the headspace of the sample and maintained at 45 °C for 30 min to adsorb volatile compounds. Subsequently, the fiber was inserted into the GC-MS injection port and desorbed at 250 °C for 5 min [22]. GC-MS analysis was performed under the following conditions: a DB-5MS capillary column (30 m × 0.25 mm × 0.25 μm) with the injector temperature at 250 °C in splitless mode. The oven temperature program was initiated at 35 °C (held for 2 min), increased to 90 °C at 5 °C/min, and then raised to 250 °C at 8 °C/min (held for 5 min). Operated in EI mode (70 eV; ion source: 250 °C), the mass spectrometer acquired spectra across 30 to 400 *m*/*z*.

Compounds were identified through retention time and mass spectra matched to NIST 14.0. (Gaithersburg, MD, USA). The relative quantification of volatile components was performed using the peak area normalization method.

### 2.5. Preparation of Longan Peel and Seed Extracts and Their Complexes with Wheat Starch

Briefly, dried longan peel powder and longan seed powder (250 g) were mixed with ethanol solution (5 L, 75%, *v*/*v*) and continuously stirred at 25 °C for 12 h, respectively. Then, the mixtures were filtered and the supernatants were condensed using an RE-501 rotary evaporator (Kankun Instrument Equipment Co., Ltd., Shanghai, China) at 45 °C, respectively. Thereafter, the longan peel extract (LPE) and longan seed extract (LSE) were obtained by freeze-drying and stored at 4 °C for further tests. The yields of LPE and LSE were 4.99% and 4.62%, respectively.

Wheat starch (5 g) was dispersed in water (80 mL) to form a suspension. LPE and LSE were added to the starch suspension at the different concentrations (0%, 5%, 10%, 15%, and 20%, *w*/*w*, based on starch), respectively. Mixtures were boiled for 30 min in boiling water with stirring until the formation of transparent paste. The gelatinized samples were cooled to 25 °C and lyophilized at −60 °C [23] and then stored at 4 °C pending further analysis.

### 2.6. Determination of Thermal Properties of Wheat Starch with LPE or LSE

The thermal properties of the sample were measured by a DSC-40A differential scanning calorimeter (Phinix Instrument, Jiangsu, China). Briefly, different concentrations (0%, 5%, 10%, 15%, or 20%, *w*/*w*, based on starch) of LPE and LSE were added to a certain amount of starch, and 3 mg of mixed powder was mixed with water (9 μL) in an aluminum crucible, respectively. After that, the aluminum crucible was sealed and equilibrated at 25 °C for 24 h and then thermally scanned from 30 to 160 °C at 10 °C/min [24]. Meanwhile, a blank crucible was used as a control. *T*_o_ (onset temperature), *T*_p_ (peak temperature), *T*_c_ (conclusion temperature), and *ΔH*_g_ (gelatinization enthalpy) were obtained.

### 2.7. Determination of Thermogravimetric Analysis (TGA) of Wheat Starch with LPE or LSE

The thermogravimetric determination of the sample was carried out by using a thermogravimetric analyzer (DGL-TGA10, DeGALLO Electronic Technology Co., Ltd., Suzhou, China). The sample (from Section 2.5) (5.0 ± 0.5 mg) was added to the aluminum crucible, sealed and incubated at 40 °C for 30 min. The heating procedure of the instrument was as follows: starting temperature 30 °C, ending temperature 440 °C, heating rate of 10 °C/min, nitrogen flow rate 20 mL/min [25], and using an empty crucible as the control. The TG curve of each sample was recorded, and the derivative thermogravimetric (DTG) curve was recorded by taking the first derivative of the TG curve.

### 2.8. Determination of Rheological Properties of Wheat Starch with LPE or LSE

The rheological properties of the sample was measured by an MCR302 rotating rheometer (Netzsch, Co., Ltd., Shanghai, China). Briefly, the wheat starch (2 g) was dispersed in water (18 mL), and then different concentrations (0%, 5%, 10%, 15%, or 20%, *w*/*w*, based on starch) of LPE and LSE were added into the starch suspension, respectively. Subsequently, the mixtures were heated in a boiling water bath for 30 min under constant agitation, and then the gelatinized starch was transferred onto the testing plate. Before the test, the sample was incubated for 5 min to achieve thermal equilibrium. Firstly, the dynamic oscillation test was carried out by amplitude scanning (0.1 to 100 Pa) at a constant frequency (1 Hz) to determine the linear viscoelastic region. Then, a dynamic frequency sweep (0.1 to 100 rad/s) was performed at 1% strain frequency, and the test temperature was set at 25 °C. Subsequently, the results of G’ (the storage modulus), G” (the loss modulus), and tanδ = G”/G’ (the loss tangent) considering the angular frequency (rad/s) were recorded.

### 2.9. Determination of Solubility and Swelling Power of Wheat Starch with LPE or LSE

The determination of this factor was performed according to the method of Yu et al. [26]. Wheat starch (1 g) was mixed with water (50 mL), and then LPE and LSE (0%, 5%, 10%, 15%, or 20%, *w*/*w*, based on the weight of starch) were added, respectively. The mixed solution was then heated in a boiling water bath for 30 min with continuous stirring and cooled to 25 °C. The sample was then centrifuged at 5000× *g* in a TG16G centrifuge for 20 min (Beihong, Henan, China), and the weight of the precipitate after centrifugation was the mass of swollen starch (*m*_1_). After centrifugation, the supernatant was transferred to an oven at 100 °C and dried to constant weight, and the mass of water-soluble starch was *m*_2_. The solubility and swelling power of the sample were calculated as follows: Solubility (%) = (*m*_2_/*m*) × 100%, and Swelling power (%) = *m*_1_/(*m* − *m*_2_) × 100%. Here, *m* is the weight of the sample/g, *m*_1_ is the weight of the swollen starch/g, and *m*_2_ is the weight of the water-soluble starch/g.

### 2.10. Determination of the Water Holding Capacity (WHC) and Oil Holding Capacity (OHC) of Wheat Starch with LPE or LSE

Firstly, different concentrations (0%, 5%, 10%, 15%, 15%, or 20%, *w*/*w*, based on starch) of LPE and LSE were added to starch for preparing the mixed powder.

Water holding capacity (WHC) was measured according to a previous study [27]. Briefly, 1 g (*m*_1_) of the mixed powder was added to water (20 mL) and heated in a boiling water bath for 30 min with continuous stirring. The mixture was then centrifuged at 8000× *g* for 10 min and the mass of the precipitate weight was m_2_. The WHC of the sample was calculated as WHC (%) = (*m*_2_ − *m*_1_)/*m*_1_.

Oil holding capacity (OHC) was measured according to a previous study [28]. Briefly, 1 g (*m*_1_) of the mixed powder was added to soybean oil (20 mL) and heated in a boiling water bath for 30 min with continuous stirring. The mixture was then centrifuged at 8000× *g* for 10 min and the mass of the precipitate weight was *m*_2_. The OHC of the sample was calculated as OHC (%) = (*m*_2_ − *m*_1_)/*m*_1_.

### 2.11. Determination of Iodine Binding of Wheat Starch with LPE or LSE

Starch (0.1 g) with different concentrations (0%, 5%, 10%, 15%, 15%, or 20%, *w*/*w*, based on starch) of LPE and LSE was added to 50 mL water, heated in a boiling water bath for 30 min with constant stirring, and placed in an ice water bath and cooled down quickly to 25 °C. The solution (2.5 mL) was mixed with 0.5 mL of iodine reagent (a mixed solution of 2% KI and 0.2% I_2_, *w*/*v*), and the mixed solution was diluted to 50 mL with water and incubated at 25 °C in the dark for 15 min. The absorbance of the mixed solution was measured in the wavelength range of 500 to 900 nm, and the iodine binding ability of the sample was reflected by the absorbance value.

### 2.12. Determination of Total Phenolic Content (TPC) and Total Flavonoid Content (TFC) of LPE and LSE

The total phenolic content of LPE and LSE were measured by the Folin–Ciocalteu method. Briefly, LPE (0.1 mL) or LSE (0.1 mL) were added to Na_2_CO_3_ solution (8.0 mL, 7.5%, *w*/*v*). Then, the Folin phenol (0.9 mL, 1 mol/L) reagent was added and the mixture was incubated at 45 °C for 30 min. The absorbance was measured at 760 nm using a UV-1800BPC spectrophotometer (Meipuada Instruments Co., Ltd., Shanghai, China) [23]. The total phenol content was calculated by a calibration curve (gallic acid as the standard): *y* = 0.68434*x* + 0.00308, *R*^2^ = 0.9931.

The total flavonoid content of LPE and LSE was measured by the colorimetric method. In brief, LPE (0.1 mL) or LSE (0.1 mL) were added water (2 mL) and NaNO_2_ solution (0.1 mL, 5%, *w*/*v*). The mixture was incubated at 25 °C for 6 min, followed by the addition of AlCl_3_ (0.2 mL, 10%, *w*/*v*). The total volume was adjusted to 3 mL with water, and the reaction was carried out at 25 °C for 5 min. The absorbance was measured at 418 nm using the UV-1800BPC spectrophotometer (Meipuada Instruments Co., Ltd., Shanghai, China) [23]. The total flavonoid content was calculated by a calibration curve (quercetin as the standard): *y* = 0.64977*x* + 0.0544, *R^2^* = 0.99923.

### 2.13. Determination of Phenolic Profile of LPE and LSE

The primary phenolic compounds in LPE and LSE were separated using ICS-6000 high-performance liquid chromatography (HPLC, Jie Dao Scientific Instruments Co., Ltd., Hangzhou, China). For the chromatographic separation, an Inertsil 3.ODS-3 column (5 μm, 4.6 × 250 mm, GL-Science, Shanghai, China) was utilized, using methanol and ultrapure water as the mobile phase. The detection wavelength was set at 240 nm with a column temperature of 25 °C. The injection volume was 20 μL, and chromatographic separation employed a binary mobile phase system consisting of methanol (A) and ultrapure water (B) containing 0.1% formic acid. The following gradient elution profile was used: 50% A (0 min), 60% A (5 min), 70% A (10 min), 80% A (15 min), 90% A (20 min), and 100% A (25 min) [29]. The HPLC-separated eluates were subsequently analyzed by mass spectrometry under the following conditions: positive ion mode with an electrospray ionization (ESI+) source, a capillary voltage of 40 eV, and a mass scan range of 40 to 1000 *m*/*z*. Compound identification was achieved by comparing retention times and mass spectral data with the NIST 14.0 database (Gaithersburg, MD, USA) and corroborated through literature analysis.

### 2.14. Determination of Antioxidant Activities of LPE and LSE

The free radical (ABTS, DPPH) scavenging activity and reducing power assays of LPE and LSE were evaluated according to a previous study by Zeng et al. [30]. LPE and LSE were dissolved in ethanol (75%, *v*/*v*) at different concentrations for measurement, respectively.

### 2.15. Statistical Analysis

Analyses were performed in triplicate for each sample, and data were expressed as mean ± standard deviation (SD). Data were analyzed by one-way ANOVA using SPSS 27.0 (SPSS Inc., Chicago, IL, USA), and Duncan’s multiple comparison test was used to determine a significant difference between each group (*p* < 0.05).

## 3. Results and Discussions

### 3.1. Texture Properties and Color of Longan Cake

As shown in Table 1, the addition of longan peel and longan seed changed the hardness of the pastry crust. With the addition of longan peel, the hardness of the pastry crust of longan cake increased from 193.14 to 348.28, and the hardness of the filling increased from 132.47 to 134.38. With the addition of longan seed, the hardness of the pastry crust in longan cake increased from 193.14 to 301.74. Previous studies report that the texture of baked products is closely associated with the starch and gluten protein content in their raw materials. Given that longan peel and longan seed are rich in polyphenols and dietary fiber, these components may interact with starch and gluten proteins during processing, thereby influencing the textural properties of the products [31]. The addition of longan peel showed a greater effect on increasing the hardness of pastry crust than the addition of longan seed. The porous and friable structure of longan peel created high-specific-surface-area particles upon pulverization, conferring exceptional water absorption capacity. When incorporated into pastry crust, longan peel immobilizes substantial amounts of moisture, thereby compromising the hydration of gluten proteins and starch. The resultant moisture competition manifests as significant textural hardening in baked products. Conversely, longan seeds exhibit smooth-surfaced particles with low hydrophilicity due to their dense, rigid matrix, consequently exerting relatively small effects on hardness enhancement. Adhesiveness refers to the work or resistance required to separate a piece of food’s surface from another material. It is typically quantified as the negative peak area in a texture analyzer force–time curve, where a higher value indicates greater adherence to contact surfaces. In addition, the adhesiveness of fillings significantly decreased from 35.80 to 29.19 and 35.80 to 27.19 with the incorporation of longan peel and longan seed to pastry crust. The minimum adhesiveness was observed in fillings at 20% longan peel and longan seed, which was attributed to moisture migration during the baking process. The high pectin content in longan peel facilitates the formation of hydrophilic gel networks that effectively immobilize moisture within the pastry crust, thereby significantly reducing water migration toward the filling. This moisture retention mechanism results in substantially less adhesiveness reduction compared to groups incorporating longan seed. Moreover, the addition of longan peel and longan seed significantly altered the color parameters of the pastry crust (a decreased L* value, increased a* value, decreased b* value and decreased *ΔE* value). Higher *ΔE* values indicate greater perceptible color differences. When *ΔE* < 1, color variance is visually undetectable to the human eye. At 1 < *ΔE* < 2.5, minute differences may be discerned by trained observers, while *ΔE* > 2.5 signifies clearly distinguishable variations to untrained individuals. As presented in Table 1, all formulations incorporating longan peel and longan seed exhibited *ΔE* > 2.5, confirming their significant effects on the chromatic properties of longan cake. The color change in longan cake pastry with the addition of longan peel and longan seed was mainly related to the color of the longan peel and longan seed themselves.

### 3.2. Volatile Flavor Components of Longan Cake

Baked products primarily develop their distinct flavors through the Maillard reaction and caramelization. Aldehydes, ketones, and furans are the dominant flavor constituents, and other compounds such as alcohols, and esters also contribute. As shown in Table 2 and Table 3, the volatile flavor profile of longan cake was predominantly composed of aldehydes, ketones, and alcohols. According to the relative content of volatile aldehydes, nonaldehyde was the most abundant aldehyde component in longan cake, followed by furfural. It has been reported that volatile aldehyde flavor components are the flavor components produced by oil oxidation and the Maillard reaction. And nonaldehyde is the product of oleic acid oxidation, which is a characteristic component of oil flavor [32]. Furfural mainly involves the high-temperature pyrolysis reaction of sugars, which provides caramelized and nutty aromas of longan cake. The experimental results showed that the addition of longan peel and longan seed increased the content of nonanal and furfural, indicating a contribution to the flavor of longan cake. The results of the relative content of volatile ketone flavor components showed that 2-heptanone and 2-nonanone were the two higher-content substances. 2-heptanone (exhibiting cheesy and fruity notes) originates from butterfat oxidation, while 2-nonanone (with creamy and coconut-like attributes) is produced via the β-oxidation of medium-chain fatty acids [33]. In addition, 2-dodecanone is degraded by long-chain fatty acids but provides less flavor activity [34]. These results showed that ketones accounted for 29 to 55% of the volatile flavor components, which were the main contributors to the flavor. The addition of longan peel and longan seed could also increase the amount of volatile ketones. 2-acetylfuran, generated through sugar pyrolysis, provides intense caramel-sweet and nutty notes even at trace concentrations, which has a certain effect on the flavor of longan cake. Alcohols in baked products predominantly stem from lipid oxidation and sugar degradation. For instance, 2-furanmethanol (caramel-sweet) was formed via furfural reduction during caramelization. But 1-octen-3-ol has a mushroom earthy and metallic flavor, which is generated by the oxidation of unsaturated fatty acids [35]. And it presents a grassy flavor at low concentration [36]. With the addition of longan peel and longan seed, the relative content of alcohols decreased, and the relative content of aldehydes increased, which indicated that they could modulate redox conditions to inhibit am aldehyde-to-alcohol reduction during lipid and sugar degradation [37].

### 3.3. Thermal Properties of Wheat Starch with LPE or LSE

The gelatinization characteristics of starch reflects its enthalpy change during the gelatinization process, where starch granules transfer from a semi-crystalline state to a disordered gel state under the combined effects of heat and water. This process significantly affects the processing properties of starch food. As shown in Table 4, the *T*_o_, *T*_p_, *T*_c_, and Δ*H*_g_ of wheat starch showed different changes with the addition of LPE and LSE. Both LPE and LSE reduced the *T*_o_, *T*_p_, *T*_c_, and Δ*H*_g_ of wheat starch, and the extent of reduction exhibited a positive correlation with their concentrations. This may be due to the abundant phenols contained in these extracts. They can physically impede interactions between starch granules. In addition, LSE showed a stronger effect on the thermal properties of wheat starch than LPE. The content of hydrophilic phenols in longan peel extracts might be low, thereby restricting their interaction with starch matrices. Furthermore, the polyphenol content in longan seed extracts might be higher than that in LPE, which might also be a factor contributing to the greater effect of LSE on enthalpy than longan peel extracts. It has been reported that phenolic compounds can facilitate starch gelatinization and lower gelatinization energy via the formation of a hydrogen bond with starch molecules [38,39,40]. Meanwhile, phenolic compounds can reduce the energy required for melting in the crystal zone by disrupting the stability of the starch crystalline region. Their phenolic hydroxyl groups can form high-strength hydrogen bonds with starch chains, replacing the original hydrogen bonds between starch molecules [41,42]. In addition, it has been reported that the embedding of small molecule phenolics in the helical cavity of amylose also leads to a decrease in the stability of the crystalline zone (a decrease in *T*_o_) and a decrease in the energy of gelatinization (a decrease in Δ*H*_g_) [19,43].

### 3.4. Thermogravimetric Analysis (TGA) of Wheat Starch with LPE or LSE

**Figure 1:**
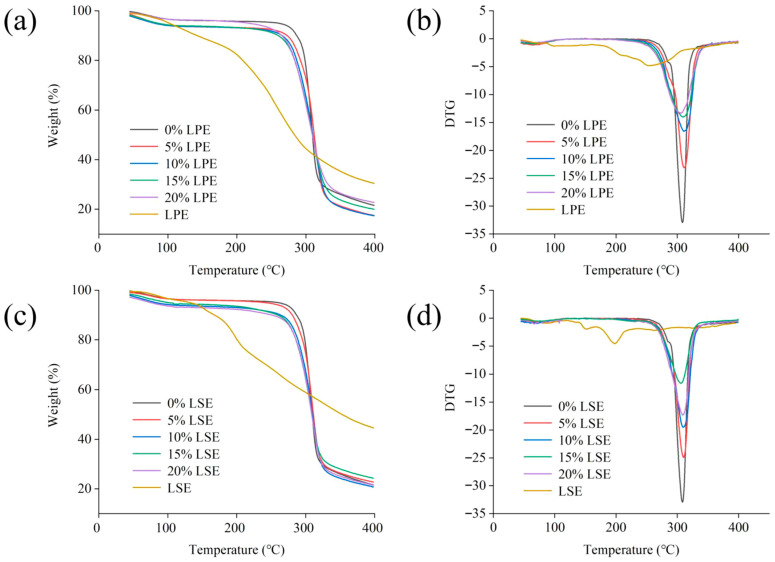
Effects of different concentrations of LPE and LSE on the TG of wheat starch: (**a**) TG curves of LPE; (**b**) DTG curves of LPE; (**c**) TG curves of LSE; (**d**) DTG curves of LSE.

As illustrated in Figure 1, the weight loss of all samples primarily occurred in two stages. During the initial stage (<120 °C), the samples exhibited a slight mass reduction due to moisture evaporation. The second stage (200 to 350 °C) displayed pronounced weight loss peaks in the DTG curves, which was attributed to the thermal decomposition of phenolic compounds and the thermal degradation of starch chains [44]. Notably, LPE commenced weight loss at 175 °C due to the thermal decomposition of polyphenols with low molecular weight [45]. LSE initiated weight loss at 150 °C, caused by the oxidation or cleavage of thermally labile components such as phenolics and saponins [45]. As shown in Figure 1, the initial decomposition temperatures for LPE groups (0%, 5%, 10%, 15% and 20% LPE) were 175 °C, 265 °C, 250 °C, 240 °C, 235 °C and 230 °C, respectively, while LSE groups (0%, 5%, 10%, 15% and 20% LSE) exhibited initial decomposition temperatures at 150 °C, 265 °C, 260 °C, 255 °C, 250 °C and 245 °C, respectively. With the addition of LPE and LSE, the initial decomposition temperature gradually decreased. Furthermore, the initial decomposition temperatures of all LPE groups (5%, 10%, 15%, and 20% LPE) were lower than those of the LSE groups (5%, 10%, 15%, 20% LSE), indicating that the thermal stability of LSE combined with wheat starch was stronger than that of LPE. LSE might contain more polyphenols that can form thermally stable coatings on starch granule surfaces, creating physical barriers and enhancing thermal degradation resistance. Furthermore, compared to pure extracts groups, starch–phenolic complexes linked via hydrogen bonds generate ‘colloid–phenolic composite shields’ with superior heat stability. In addition, some phenols in extracts undergo oxidative decomposition at 150 °C, generating free radicals that attack starch chains. Thermal degradation products (e.g., aldehydes, ketones) additionally catalyze starch breaking. Although LPE exhibited weaker effects than LSE, its phenols might still bind with starch so as to affect the thermal properties of starch. The initial decomposition temperature of the LPE and LSE groups was lower than that of the control group (265 °C), indicating that the formation of the complex might inhibit the thermal degradation of phenolic substances and reduce the stability of starch. These phenomena may be related to the formation of hydrogen bonds between phenolics and starch [46].

### 3.5. Rheological Properties of Wheat Starch with LPE or LSE

**Figure 2:**
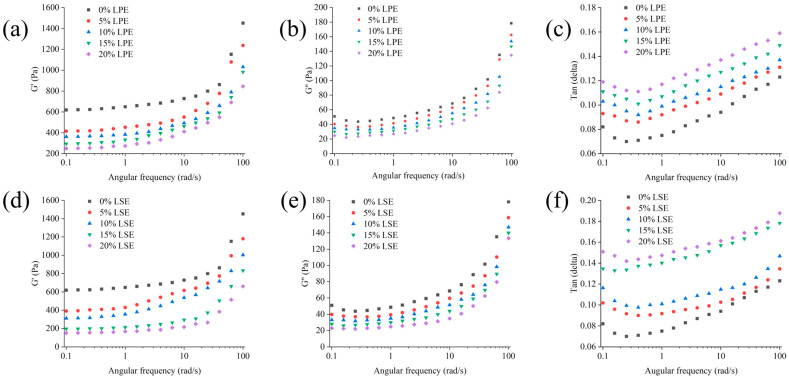
Effects of different concentrations of LPE and LSE on the rheological properties of wheat starch: (**a**) G’ of LPE; (**b**) G’’ of LPE; (**c**) tanδ of LPE; (**d**) G’ of LSE; (**e**) G’’ of LSE; (**f**) tanδ of LSE.

The effects of LPE and LSE on the rheological properties of wheat starch were illustrated in Figure 2. With the addition of LSE and LPE, the G’ values of starch were reduced, while the reduction in LSE groups was greater than that of LPE groups. The trend in G’’ values was similar to that of G’ values, and both G’’ and G’ values decreased with the addition of LPE and LSE, which showed a concentration-dependent effect. In addition, both LPE and LSE increased the tanδ value of starch. It has been reported that tanδ indicates the solid-like or liquid-like behavior of a material. A lower tanδ corresponds to a more rigid, elastic-dominant structure, while a higher tanδ signifies a shift toward liquid-like behavior [47]. In the low-frequency regime, slower deformation rates allow sufficient time for structural reorganization, resulting in viscous flow dominance. Conversely, under high-frequency deformation, the rapid strain rate precludes adequate structural relaxation, leading to enhanced elastic deformation. The tanδ of LPE was greater than that of LSE, indicating that the LSE group had a more stable structure, while the LPE group had greater fluidity. This also shows that the LSE group has a higher proportion of elastic components and stronger solid-like behavior. LPE might contain significantly fewer lipophilic phenols than LSE, resulting in minimal effective plasticizer concentration. Furthermore, hydrophilic phenols in LPE form rigid gel matrices with starch that sterically hinder phenol diffusion. Conversely, lipophilic phenols in LSE perform as an endogenous lubricant, synergistically enhancing phenolic-mediated plasticization with starch via hydrophobic interactions. All present results demonstrated that both LPE and LSE could promote a transition of starch from solid-like to liquid-like behavior. Phenols in LPE and LSE competitively could bind to hydrogen bonding sites in starch chains [48], disrupting the starch network. Previous studies show that phenols destabilize the non-crystalline region [49], weakening the rigid starch matrix and reducing G’ values. It has been reported that phenols in plant extracts can be incorporated into amylose helical cavities, forming V-type single-helical crystals [50]. This disrupts native A-type double-helical structures, which may shorten effective starch chain lengths and enhance molecular mobility, thereby increasing tanδ.

### 3.6. Solubility and Swelling Power of Wheat Starch with LPE or LSE

**Figure 3:**
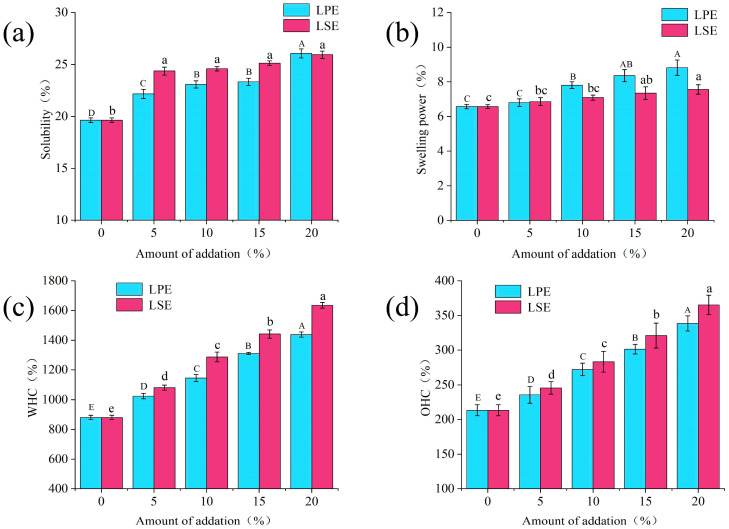
Effects of LPE and LSE on the water holding capacity, oil holding capacity, solubility and swelling power of wheat starch: (**a**) solubility; (**b**) swelling power; (**c**) WHC; (**d**) OHC.

The solubility and swelling power of starch reflect the density of its structure and the strength of its crystalline zone. As shown in Figure 3a,b, the addition of LPE and LSE significantly enhanced the solubility and swelling power of wheat starch, which showed a concentration-dependent effect. With the addition of LPE and LSE, the solubility increased from 19.63% to 26.07% and 25.94%, respectively. With the exception of the 20% addition group, LSE groups exhibited significantly higher solubility than the LPE groups. With the addition of LPE and LSE, the swelling power increased from 6.57% to 8.82% and 7.56%, respectively. Moreover, swelling power was consistently greater in some LPE groups compared to LSE groups. Some hydrophilic phenols in extracts may intercalate into amorphous domains of starch granules, expanding intermolecular d-spacing and facilitating water molecule penetration. Concurrently, lipophilic phenols in extracts may reduce water–starch interfacial tension, thereby enhancing hydration kinetics. The literature reports indicate that phenolic hydroxyl groups of phenols compete with starch chains for binding sites, disrupting intermolecular hydrogen bonds and facilitating water molecule penetration. Additionally, phenolic compounds can weaken the binding strength within starch’s crystalline lamellae, promoting water infiltration into starch granules and consequently improving solubility and swelling capacity [26]. These findings collectively demonstrated that phenols effectively modify starch functional properties by enhancing solubility and swelling power, thereby potentially modifying the quality attributes of starch products.

### 3.7. Water Holding Capacity (WHC) and Oil Holding Capacity (OHC) of Wheat Starch with LPE or LSE

WHC and OHC are critical properties of starch foods, significantly affecting the quality and sensory attributes of products. As shown in Figure 3b,d, the addition of LPE and LSE significantly increased both the WHC and OHC of wheat starch in a concentration-dependent manner. With the addition of LPE and LSE, WHC increased from 880.61% to 1438.31% and 1635.13%, respectively. With the addition of LPE and LSE, OHC increased from 213.34% to 338.52% and 365.37%, respectively. Regarding WHC, the phenolic compounds present in LSE and LPE form novel hydrogen bonding networks with starch molecules, effectively entrapping water and reducing moisture migration, thereby enhancing WHC. For OHC, hydrophobic interactions between these phenolic compounds and fatty acid chains facilitate the formation of ‘starch–polyphenol–lipid’ ternary complexes, thereby enhancing OHC [29]. Notably, the addition of LSE more strongly enhanced WHC and OHC than LPE. Consequently, the LSE group conferred significantly greater improvements in WHC and OHC than LPE. LSE might have higher total phenol content than LPE, which would enhance its penetration into starch amorphous domains, thereby increasing the hydration volume. In addition, LSE might have a higher content of lipophilic phenols than LPE, thus facilitating lipid–phenolic synergism and enhancing WHC and OHC. These dual effects collectively endow LSE with superior starch hydration and oil retention capabilities than LPE.

### 3.8. Iodine Binding of Wheat Starch with LPE or LSE

The maximum absorption wavelength (λ_max_) directly reflects the helical conformation integrity of amylose in starch. As shown in Table 5, the addition of LPE and LSE both induced a blue shift in the λ_max_ of wheat starch, which suggested that the helical structure of amylose was disrupted. This result was attributed to the interaction between the polyphenols and starch. In addition, the helix length of the amylose–iodine complex was shortened [51]. The A_635_ value usually reflects the concentration of the iodine–starch complex. The decrease in the A_635_ value of LPE and LSE groups indicated that the reaction of wheat starch with LPE and LSE led to a decrease in the concentration of the iodine–starch complex. This was attributed to the presence of competitive binding between the straight-chain amylose of wheat starch and the polyphenols in LPE and LSE, occupying the iodine–starch binding site. Commonly, alterations in the aggregation state of iodine ions within the helical cavities of starch are typically reflected by the magnitude of the A_635_/A_520_ ratio. With the addition of LPE and LSE, this ratio progressively decreased, suggesting the disordering of the iodine clusters within the starch helices. Furthermore, an analysis of the three iodine binding indicators revealed significantly greater deviation in the LSE group than the LPE group. This demonstrates that LSE exerts a more pronounced effect on starch’s iodine binding capacity than LPE, which might be attributable to its high content of phenols.

### 3.9. Total Phenolic and Total Flavonoid Content of LPE and LSE

By using Folin–Ciocalteu and AlCl_3_ colormetric methods, the total phenolic content and total flavonoid content were calculated as 71.05 ± 2.57 mg/g (based on gallic acid) and 286.27 ± 6.04 mg/g (based on quercetin) for LPE and 78.49 ± 5.21 mg/g (based on gallic acid) and 423.21 ± 7.69 (based on quercetin) for LSE, respectively.

### 3.10. Identification of Main Phenolic Components in LPE and LSE

**Figure 4:**
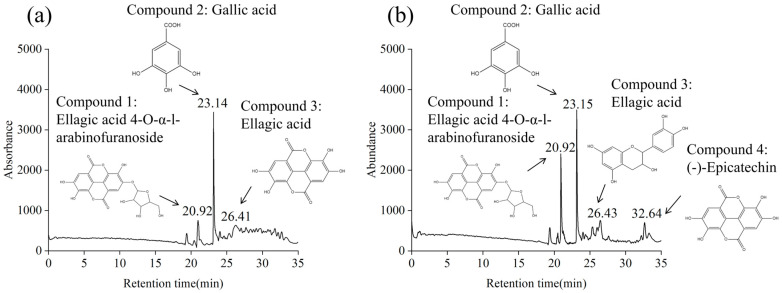
Identification of main phenolic compounds of LPE and LSE: (**a**) LPE; (**b**) LSE.

As expected, both LPE and LSE were demonstrated to contain high levels of polyphenols. Consequently, the main phenolic compounds in LPE and LSE were further identified using HPLC-MS. As shown in Figure 4, according to HPLC separation, three major phenolic compounds were detected from LPE, and the MS of three material components resulted in the [M+H]^+^ at *m*/*z* 435.04, 171.02 and 303.1, indicating the molecular weight of 434, 170 and 302. After a comparison, the major phenolic components in LPE were ellagic acid 4-O-α-l-arabinofuranoside, gallic acid and ellagic acid, respectively [52]. Four main phenolic compounds were also detected in LSE, which resulted in the [M+H]^+^ at *m*/*z* 435.04, 171.02, 303.1 and 291.2, indicating the molecular weight of 434, 170, 302 and 290. The main phenolic components in LSE were ellagic acid 4-O-α-L-arabinofuranoside, gallic acid, ellagic acid, and (-) -epicatechin [53].

### 3.11. Antioxidant Activities of LPE and LSE

**Figure 5:**
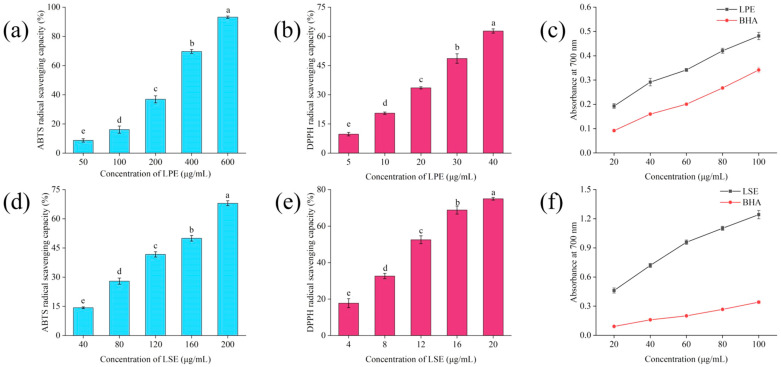
Antioxidant capacity of LPE and LSE: (**a**) ABTS radical scavenging activity of LPE; (**b**) DPPH radical scavenging activity of LPE; (**c**) reducing power of LPE; (**d**) ABTS radical scavenging activity of LSE; (**e**) DPPH radical scavenging activity of LSE; (**f**) reducing power of LSE.

As illustrated in Figure 5, LPE exhibited a concentration-dependent increase in ABTS radical scavenging activity, with an EC_50_ value of 301.37 ± 7.34 μg/mL. In comparison, LSE displayed a better scavenging activity, with ABTS radical scavenging activity increasing from 14.63 ± 0.47% to 68.03 ± 1.26% and a significantly lower EC_50_ of 145.09 ± 3.41 μg/mL, indicating superior efficiency in scavenging ABTS radicals. For DPPH radical scavenging, LPE demonstrated a concentration-dependent increase in DPPH radical scavenging activity, with an EC_50_ value of 31.07 ± 0.21 μg/mL. And LSE exhibited an even stronger scavenging capacity, with an EC_50_ value of 12.17 ± 0.16 μg/mL. The reducing power revealed that both LPE and LSE surpassed the BHA at equivalent concentrations, confirming their good reducing power. These results were attributed to their high content of phenolic and flavonoid compounds. Recent studies further indicate that phenolic compounds can significantly improve the antioxidant capacity of starch foods [18,38].

## 4. Conclusions

This study investigated the effects of longan peel and seed on the quality of longan cake. In addition, the effects of longan peel extract (LPE) and longan seed extract (LSE) on the physicochemical properties of wheat starch were also determined. The results showed that longan peel and seed affected the color, texture, and volatile flavor profile of longan cake, enriching its sensory characteristics with more diverse aroma compounds. The results demonstrated that LSE had a stronger effect than LPE on the gelatinization characteristics, rheological characteristics, solubility, water/oil retention capacity, and iodine binding capacity of starch, while LPE had a greater effect than LSE on the thermogravimetric analysis and swelling power of starch. Furthermore, except for thermogravimetric analysis, the effects of LSE were stronger than those of LPE. Component analysis indicated that LPE and LSE contained abundant phenolic compounds, which contributed to their remarkable antioxidant activity and interaction with starch. Both LPE and LSE had high contents of total phenols (LPE: 71.05 ± 2.57 mg/g, LSE: 78.49 ± 5.21 mg/g) and total flavonoids (LPE: 286.27 ± 6.04 mg/g, LSE: 423.21 ± 7.69 mg/g). The present study suggests that longan peel and seed exhibit great potential as natural additives for improving the quality of wheat starch products. However, a sensory evaluation and analysis of their applicability in other types of starch foods are still needed. A further study is currently underway to assess the applications of these longan by-products in different starch food (such as biscuits and bread) and their functional benefits.

## Figures and Tables

**Table 1:** Effects of longan peel and seed on the color and texture of longan cake.

Sample		Texture Profile Analysis			Color			
		Pastry Hardness (g)	Filling Hardness (g)	Filling Adhesiveness	L*	a*	b*	*ΔE*
longan peel	0%	193.14 ± 10.79 ^c^	132.47 ± 1.67 ^b^	35.80 ± 0.48 ^a^	63.92 ± 2.27 ^a^	9.60 ± 0.40 ^e^	43.13 ± 1.30 ^a^	-
	5%	229.84 ± 15.17 ^bc^	132.52 ± 1.39 ^b^	34.04 ± 0.53 ^ab^	56.96 ± 0.61 ^b^	11.10 ± 0.81 ^d^	40.02 ± 0.66 ^b^	7.01 ± 0.46 ^d^
	10%	272.14 ± 17.46 ^b^	133.35 ± 1.42 ^a^	33.21 ± 1.24 ^bc^	55.12 ± 0.90 ^c^	12.08 ± 0.75 ^c^	38.68 ± 1.08 ^c^	9.43 ± 0.58 ^c^
	15%	288.24 ± 14.56 ^ab^	133.44 ± 0.99 ^a^	31.45 ± 0.47 ^c^	53.22 ± 1.56 ^d^	13.18 ± 0.41 ^b^	35.90 ± 0.96 ^d^	12.72 ± 0.73 ^b^
	20%	348.28 ± 10.03 ^a^	134.38 ± 1.34 ^a^	29.19 ± 1.58 ^d^	52.18 ± 1.38 ^d^	14.16 ± 0.96 ^a^	35.36 ± 0.98 ^d^	14.13 ± 0.63 ^a^
longan seed	0%	193.14 ± 10.79 ^c^	132.47 ± 1.67 ^b^	35.80 ± 0.48 ^a^	63.92 ± 2.27 ^a^	9.60 ± 0.40 ^b^	43.13 ± 1.30 ^a^	-
	5%	210.86 ± 11.74 ^bc^	136.73 ± 1.21 ^a^	33.27 ± 0.57 ^b^	57.84 ± 0.81 ^b^	10.84 ± 0.90 ^a^	34.08 ± 0.80 ^b^	10.52 ± 0.23 ^d^
	10%	247.26 ± 8.73 ^b^	130.43 ± 1.75 ^bc^	31.30 ± 0.94 ^c^	54.06 ± 1.54 ^c^	11.36 ± 0.56 ^a^	33.26 ± 1.03 ^bc^	13.47 ± 0.49 ^c^
	15%	254.12 ± 18.09 ^ab^	134.47 ± 1.63 ^ab^	28.60 ± 0.72 ^cd^	50.70 ± 1.50 ^d^	11.50 ± 0.23 ^a^	32.44 ± 0.34 ^c^	16.45 ± 0.68 ^b^
	20%	301.74 ± 10.72 ^a^	129.48 ± 1.07 ^c^	27.19 ± 1.07 ^d^	47.62 ± 0.50 ^d^	11.52 ± 0.56 ^a^	29.14 ± 0.46 ^d^	20.92 ± 0.85 ^a^

Different lowercase letters in each vertical column among the same sample group (longan peel group and longan seed group) denote statistically significant differences (*p* < 0.05).

**Table 2:** Effects of longan peel and seed on the volatile substance content of longan cake.

RT (min)	Compounds	Volatile Compound Contents (%)									
		Longan Peel					Longan Seed				
		0%	5%	10%	15%	20%	0%	5%	10%	15%	20%
16.511	2-Heptanone	28.24 ± 0.03 ^b^	22.25 ± 0.02 ^d^	20.83 ± 0.02 ^f^	22.70 ± 0.02 ^c^	20.67 ± 0.02 ^g^	28.24 ± 0.03 ^b^	29.53 ± 0.03 ^a^	21.77 ± 0.02 ^e^	17.02 ± 0.02 ^i^	19.45 ± 0.02 ^h^
17.026	UN. Decane	N. D.	N. D.	N. D.	N. D.	N. D.	N. D.	2.31 ± 0.01 ^c^	2.60 ± 0.01 ^b^	N. D.	3.19 ± 0.01 ^a^
17.066	Dodecane	1.47 ± 0.01 ^c^	3.19 ± 0.01 ^a^	2.53 ± 0.01 ^b^	1.04 ± 0.01 ^e^	1.34 ± 0.01 ^d^	1.47 ± 0.01 ^c^	N. D.	N. D.	0.91 ± 0.01 ^f^	N. D.
17.755	2-Octyne	N. D.	0.87 ± 0.01 ^d^	N. D.	N. D.	N. D.	N. D.	N. D.	13.25 ± 0.02 ^c^	22.33 ± 0.02 ^a^	20.94 ± 0.02 ^b^
17.765	2-Heptyne	N. D.	N. D.	N. D.	1.57 ± 0.01 ^b^	1.54 ± 0.01 ^b^	N. D.	29.53 ± 0.03 ^a^	N. D.	N. D.	N. D.
18.167	1-Pentanol	2.21 ± 0.01 ^b^	2.20 ± 0.01 ^b^	2.14 ± 0.01 ^c^	2.92 ± 0.01 ^a^	2.14 ± 0.01 ^c^	2.21 ± 0.01 ^b^	1.78 ± 0.01 ^d^	1.67 ± 0.01 ^f^	N. D.	1.59 ± 0.01 ^e^
19.390	2-Propanone, 1-methoxy-	N. D.	N. D.	N. D.	0.53 ± 0.01 ^a^	0.53 ± 0.01 ^a^	N. D.	N. D.	N. D.	N. D.	N. D.
19.550	Octanal	0.92 ± 0.01 ^f^	1.49 ± 0.01 ^c^	1.77 ± 0.01 ^a^	1.52 ± 0.01 ^b^	1.27 ± 0.01 ^d^	0.92 ± 0.01 ^f^	0.93 ± 0.01 ^f^	0.77 ± 0.01 ^g^	N. D.	1.14 ± 0.01 ^e^
19.800	Octane, 4-methyl-	0.75 ± 0.01 ^c^	1.12 ± 0.01 ^a^	1.01 ± 0.01 ^b^	0.66 ± 0.01 ^d^	N. D.	0.75 ± 0.01 ^c^	0.58 ± 0.01 ^e^	0.50 ± 0.01 ^f^	N. D.	0.49 ± 0.01 ^f^
19.804	2-Propanone, 1-hydroxy-	N. D.	N. D.	N. D.	N. D.	0.92 ± 0.01 ^a^	N. D.	N. D.	N. D.	N. D.	N. D.
20.052	Prenol	N. D.	0.65 ± 0.01 ^c^	0.67 ± 0.01 ^c^	1.05 ± 0.01 ^b^	1.18 ± 0.01 ^a^	N. D.	N. D.	N. D.	N. D.	N. D.
20.224	2-Cyclopenten-1-one, 3-methyl-	N. D.	N. D.	N. D.	N. D.	N. D.	N. D.	N. D.	0.89 ± 0.01 ^c^	1.09 ± 0.01 ^b^	1.37 ± 0.01 ^a^
20.320	2,3-Octanedione	N. D.	N. D.	N. D.	N. D.	0.33 ± 0.01 ^a^	N. D.	N. D.	N. D.	N. D.	N. D.
20.491	2-Heptenal, (Z)-	0.56 ± 0.01 ^g^	1.28 ± 0.01 ^b^	1.20 ± 0.01 ^c^	1.20 ± 0.01 ^c^	1.35 ± 0.01 ^a^	0.56 ± 0.01 ^g^	0.39 ± 0.01 ^h^	0.72 ± 0.01 ^e^	0.64 ± 0.01 ^f^	0.86 ± 0.01 ^d^
20.716	5-Hepten-2-one, 6-methyl-	0.84 ± 0.01 ^c^	0.87 ± 0.01 ^b^	0.89 ± 0.01 ^a^	0.88 ± 0.01 ^ab^	0.71 ± 0.01 ^d^	0.84 ± 0.01 ^c^	0.51 ± 0.01 ^g^	0.58 ± 0.01 ^f^	0.72 ± 0.01 ^d^	0.64 ± 0.01 ^e^
20.826	1-Hexanol	7.33 ± 0.01 ^a^	6.41 ± 0.01 ^b^	5.92 ± 0.01 ^e^	6.38 ± 0.01 ^c^	4.54 ± 0.01 ^i^	7.33 ± 0.01 ^a^	6.35 ± 0.01 ^d^	5.08 ± 0.01 ^h^	5.51 ± 0.01 ^f^	5.12 ± 0.01 ^g^
21.978	2-Nonanone	15.41 ± 0.02 ^a^	0.32 ± 0.01 ^h^	10.56 ± 0.02 ^d^	9.89 ± 0.01 ^f^	10.38 ± 0.01 ^e^	15.41 ± 0.02 ^a^	11.31 ± 0.02 ^b^	10.87 ± 0.02 ^c^	10.87 ± 0.02 ^c^	8.80 ± 0.01 ^g^
22.113	Nonanal	10.21 ± 0.02 ^f^	17.79 ± 0.02 ^a^	14.95 ± 0.02 ^b^	13.02 ± 0.02 ^c^	11.09 ± 0.02 ^e^	10.21 ± 0.02 ^f^	7.92 ± 0.01 ^h^	7.41 ± 0.01 ^i^	11.22 ± 0.02 ^d^	10.10 ± 0.02 ^g^
23.012	1-Octen-3-ol	1.55 ± 0.01 ^g^	1.77 ± 0.01 ^c^	1.70 ± 0.01 ^e^	1.68 ± 0.01 ^e^	1.74 ± 0.01 ^d^	1.55 ± 0.01 ^g^	1.40 ± 0.01 ^h^	1.64 ± 0.01 ^f^	1.97 ± 0.01 ^b^	2.03 ± 0.01 ^a^
23.163	Methoxyacetic acid, hexyl ester	1.22 ± 0.01 ^b^	1.77 ± 0.01 ^a^	N. D.	N. D.	N. D.	1.22 ± 0.01 ^b^	N. D.	N. D.	N. D.	N. D.
23.163	Acetic acid	N. D.	N. D.	1.86 ± 0.01 ^c^	1.90 ± 0.01 ^b^	2.43 ± 0.01 ^a^	N. D.	N. D.	1.18 ± 0.01 ^f^	1.69 ± 0.01 ^d^	1.37 ± 0.01 ^e^
23.169	1-Hexanol, 3-methyl-	N. D.	N. D.	N. D.	N. D.	N. D.	N. D.	0.74 ± 0.01 ^a^	N. D.	N. D.	N. D.
23.617	Furfural	2.12 ± 0.01 ^f^	3.72 ± 0.01 ^d^	3.96 ± 0.01 ^c^	4.34 ± 0.01 ^b^	6.32 ± 0.01 ^a^	2.12 ± 0.01 ^f^	2.05 ± 0.01 ^g^	2.14 ± 0.01 ^f^	2.93 ± 0.01 ^e^	1.90 ± 0.01 ^h^
23.856	2-Ethyl-1-hexanol	0.86 ± 0.01 ^d^	1.14 ± 0.01 ^b^	0.66 ± 0.01 ^i^	0.94 ± 0.01 ^c^	0.81 ± 0.01 ^e^	0.86 ± 0.01 ^d^	0.72 ± 0.01 ^gh^	0.73 ± 0.01 ^fg^	1.34 ± 0.01 ^a^	0.71 ± 0.01 ^h^
24.515	Ethanone, 1-(2-furanyl)-	N. D.	2.04 ± 0.01 ^a^	1.07 ± 0.01 ^d^	1.31 ± 0.01 ^c^	1.71 ± 0.01 ^b^	N. D.	N. D.	0.33 ± 0.01 ^e^	N. D.	N. D.
24.576	Copaene	N. D.	N. D.	1.08 ± 0.01 ^c^	1.21 ± 0.01 ^b^	1.48 ± 0.01 ^a^	N. D.	N. D.	0.71 ± 0.01 ^f^	0.89 ± 0.01 ^e^	0.94 ± 0.01 ^d^
24.771	3,5-Octadien-2-one	N. D.	N. D.	0.42 ± 0.01 ^bc^	N. D.	0.33 ± 0.01 ^e^	N. D.	N. D.	0.46 ± 0.01 ^a^	0.40 ± 0.01 ^d^	0.41 ± 0.01 ^cd^
25.406	Benzaldehyde	0.23 ± 0.01 ^c^	N. D.	0.24 ± 0.01 ^c^	N. D.	0.41 ± 0.01 ^b^	0.23 ± 0.01 ^c^	N. D.	N. D.	0.60 ± 0.01 ^a^	N. D.
25.183	1-Nonanol	1.29 ± 0.01 ^a^	N. D.	N. D.	N. D.	N. D.	1.29 ± 0.01 ^a^	N. D.	N. D.	N. D.	N. D.
25.186	1-Octanol	N. D.	2.12 ± 0.01 ^a^	2.00 ± 0.01 ^b^	1.76 ± 0.01 ^c^	1.74 ± 0.01 ^d^	N. D.	1.32 ± 0.01 ^e^	N. D.	N. D.	N. D.
25.19	2-Nonenal, (E)-	N. D.	N. D.	N. D.	N. D.	N. D.	N. D.	N. D.	1.50 ± 0.01 ^c^	2.12 ± 0.01 ^a^	2.02 ± 0.01 ^b^
26.265	2-Undecanone	4.73 ± 0.01 ^a^	4.70 ± 0.01 ^b^	3.66 ± 0.01 ^d^	3.25 ± 0.01 ^g^	3.27 ± 0.01 ^f^	4.73 ± 0.01 ^a^	2.93 ± 0.01 ^h^	3.37 ± 0.01 ^e^	4.11 ± 0.01 ^c^	2.61 ± 0.01 ^i^
26.445	Ethanol, 2-(2-ethoxyethoxy)-	0.07 ± 0.01 ^f^	0.60 ± 0.01 ^a^	0.23 ± 0.01 ^c^	N. D.	N. D.	0.07 ± 0.01 ^f^	0.25 ± 0.01 ^b^	0.12 ± 0.01 ^d^	N. D.	0.10 ± 0.01 ^e^
26.560	Butanoic acid	0.39 ± 0.01 ^i^	1.28 ± 0.01 ^a^	1.07 ± 0.01 ^c^	1.18 ± 0.01 ^b^	0.98 ± 0.01 ^d^	0.39 ± 0.01 ^i^	0.54 ± 0.01 ^h^	0.64 ± 0.01 ^g^	0.89 ± 0.01 ^e^	0.76 ± 0.01 ^f^
26.713	Bicyclo [7.2.0]undec-4-ene, 4,11,11-trimethyl-8-methylene-,[1R-(1R*,4Z,9S*)]-	N. D.	N. D.	1.76 ± 0.01 ^b^	1.09 ± 0.01 ^d^	1.67 ± 0.01 ^c^	N. D.	0.72 ± 0.01 ^e^	N. D.	N. D.	3.09 ± 0.01 ^a^
26.718	Caryophyllene	N. D.	1.26 ± 0.01 ^a^	N. D.	N. D.	N. D.	N. D.	N. D.	N. D.	N. D.	N. D.
27.091	2-Furanmethanol	11.16 ± 0.02 ^a^	5.63 ± 0.01 ^e^	5.19 ± 0.01 ^f^	4.38 ± 0.01 ^h^	5.01 ± 0.01 ^g^	11.16 ± 0.02 ^a^	8.07 ± 0.01 ^b^	6.92 ± 0.01 ^c^	5.77 ± 0.01 ^d^	4.72 ± 0.01 ^i^
27.257	Benzeneacetaldehyde	0.97 ± 0.01 ^h^	1.51 ± 0.01 ^f^	1.85 ± 0.01 ^d^	1.91 ± 0.01 ^c^	2.11 ± 0.01 ^a^	0.97 ± 0.01 ^h^	1.02 ± 0.01 ^g^	1.72 ± 0.01 ^e^	2.10 ± 0.01 ^a^	1.96 ± 0.01 ^b^
28.021	Humulene	N. D.	N. D.	N. D.	N. D.	N. D.	N. D.	N. D.	0.40 ±	0.62 ±	0.76 ±
28.025	Linalyl acetate	N. D.	N. D.	N. D.	0.47 ± 0.01 ^b^	0.73 ± 0.01 ^a^	N. D.	N. D.	N. D.	N. D.	N. D.
29.296	Naphthalene, 1,2,3,5,6,8a-hexahydro-4,7-dimethyl-1-(1-methylethyl)-, (1S-cis)-	N. D.	N. D.	N. D.	N. D.	0.22 ± 0.01 ^b^	N. D.	N. D.	N. D.	0.27 ± 0.01 ^a^	0.27 ± 0.01 ^a^
29.402	1-Dodecanol, 3,7,11-trimethyl-	N. D.	0.26 ± 0.01 ^a^	N. D.	N. D.	N. D.	N. D.	N. D.	0.15 ± 0.01 ^c^	0.14 ± 0.01 ^c^	0.17 ± 0.01 ^b^
29.829	2-Tridecanone	0.84 ± 0.01 ^c^	0.93 ± 0.01 ^a^	0.72 ± 0.01 ^d^	0.54 ± 0.01 ^g^	0.56 ± 0.01 ^f^	0.84 ± 0.01 ^c^	0.50 ± 0.01 ^h^	0.68 ± 0.01 ^e^	0.88 ± 0.01 ^b^	0.51 ± 0.01 ^h^
29.992	2,4-Decadienal	N. D.	N. D.	N. D.	N. D.	N. D.	N. D.	N. D.	N. D.	0.37 ± 0.01 ^a^	0.32 ± 0.01 ^b^
30.145	Hexanoic acid	0.26 ± 0.01 ^g^	1.58 ± 0.01 ^b^	1.61 ± 0.01 ^a^	1.46 ± 0.01 ^c^	1.36 ± 0.01 ^d^	0.26 ± 0.01 ^g^	0.61 ± 0.01 ^f^	1.09 ± 0.01 ^e^	1.58 ± 0.01 ^b^	1.46 ± 0.01 ^c^
31.370	Butylated Hydroxytoluene	6.37 ± 0.01 ^g^	11.25 ± 0.02 ^a^	7.46 ± 0.01 ^e^	8.95 ± 0.01 ^d^	9.13 ± 0.01 ^c^	6.37 ± 0.01 ^g^	7.08 ± 0.01 ^f^	9.92 ± 0.02 ^b^	N. D.	N. D.
33.239	Octanoic acid	N. D.	N. D.	0.54 ± 0.01 ^b^	0.27 ± 0.01 ^c^	N. D.	N. D.	N. D.	0.19 ± 0.01 ^e^	1.02 ± 0.01 ^a^	0.21 ± 0.01 ^d^

Different lowercase letters in each parallel row among the same sample group (the longan peel group and longan seed group) denote statistically significant differences (*p* < 0.05). The abbreviation ‘N. D.’ means ‘not determined’.

**Table 3:** Effects of longan peel and seed on the species and quantity of volatile substances of longan cake.

Sample			Aldehydes	Ketones	Alcohols	Acids	Esters	Furans	Phenols	Total Content
longan peel	0%	amount	6	5	6	3	N. D.	N. D.	1	21
		content (%)	15.01 ± 0.07	50.06 ± 0.08	24.40 ± 0.07	1.87 ± 0.03	N. D.	N. D.	6.37 ± 0.01	97.71 ± 0.26
	5%	amount	5	5	8	3	N. D.	1	1	23
		content (%)	25.79 ± 0.06	29.07 ± 0.06	20.18 ± 0.08	4.63 ± 0.03	N. D.	2.04 ± 0.01	11.25 ± 0.02	92.96 ± 0.26
	10%	amount	6	6	6	3	N. D.	1	1	23
		content (%)	23.97 ± 0.07	37.08 ± 0.08	16.28 ± 0.06	4.54 ± 0.03	N. D.	1.07 ± 0.01	7.46 ± 0.01	90.4 ± 0.26
	15%	amount	5	6	7	4	1	1	1	25
		content (%)	21.99 ± 0.06	37.79 ± 0.07	19.11 ± 0.07	4.81 ± 0.04	0.47 ± 0.01	1.31 ± 0.01	8.95 ± 0.01	94.43 ± 0.27
	20%	amount	6	9	7	3	1	1	1	28
		content (%)	22.55 ± 0.07	37.70 ± 0.10	17.16 ± 0.07	4.77 ± 0.03	0.73 ± 0.01	1.71 ± 0.01	9.13 ± 0.01	93.75 ± 0.30
longan seed	0%	amount	6	5	6	3	N. D.	N. D.	1	21
		content (%)	15.01 ± 0.07	50.06 ± 0.08	24.40 ± 0.07	1.87 ± 0.03	N. D.	N. D.	6.37 ± 0.01	97.71 ± 0.26
	5%	amount	5	5	7	2	N. D.	N. D.	1	20
		content (%)	12.31 ± 0.05	44.78 ± 0.08	20.38 ± 0.07	1.15 ± 0.02	N. D.	N. D.	7.08 ± 0.01	85.7 ± 0.23
	10%	amount	6	7	6	4	N. D.	1	1	25
		content (%)	14.26 ± 0.06	38.62 ± 0.09	16.19 ± 0.06	3.10 ± 0.04	N. D.	0.33 ± 0.01	9.92 ± 0.02	82.42 ± 0.28
	15%	amount	7	7	5	4	N. D.	N. D.	N. D.	23
		content (%)	19.98 ± 0.08	35.09 ± 0.09	14.73 ± 0.05	5.18 ± 0.04	N. D.	N. D.	N. D.	74.92 ± 0.26
	20%	amount	7	7	6	4	N. D.	N. D.	N. D.	24
		content (%)	18.30 ± 0.08	33.79 ± 0.08	14.34 ± 0.06	3.80 ± 0.04	N. D.	N. D.	N. D.	70.23 ± 0.26

The abbreviation ‘N. D.’ means ‘not determined’.

**Table 4:** Effects of LPE and LSE on the thermal properties of wheat starch.

Sample		DSC			
		*T*_o_ (°C)	*T*_P_ (°C)	*T*_C_ (°C)	*ΔH*g (J/g)
LPE	0%	56.54 ± 0.25 ^a^	65.73 ± 0.32 ^a^	62.90 ± 0.42 ^a^	3.34 ± 0.02 ^a^
	5%	56.16 ± 0.34 ^ab^	65.63 ± 0.79 ^ab^	62.57 ± 0.45 ^ab^	3.10 ± 0.08 ^b^
	10%	56.13 ± 0.38 ^b^	65.02 ± 0.36 ^b^	62.23 ± 0.19 ^bc^	2.94 ± 0.10 ^bc^
	15%	55.60 ± 0.84 ^c^	64.17 ± 0.68 ^c^	61.88 ± 0.16 ^cd^	2.92 ± 0.02 ^c^
	20%	55.18 ± 0.41 ^d^	64.05 ± 0.43 ^c^	61.78 ± 0.04 ^d^	2.84 ± 0.10 ^c^
LSE	0%	56.54 ± 0.25 ^a^	65.73 ± 0.32 ^a^	62.90 ± 0.42 ^a^	3.34 ± 0.02 ^a^
	5%	56.13 ± 0.54 ^b^	65.55 ± 0.28 ^ab^	62.18 ± 0.37 ^b^	2.81 ± 0.02 ^b^
	10%	56.06 ± 0.60 ^c^	64.12 ± 0.15 ^b^	62.08 ± 0.28 ^c^	2.75 ± 0.08 ^bc^
	15%	55.52 ± 0.21 ^d^	63.23 ± 0.18 ^c^	61.85 ± 0.32 ^cd^	2.59 ± 0.07 ^c^
	20%	55.08 ± 0.21 ^e^	63.02 ± 0.16 ^c^	61.48 ± 0.46 ^d^	2.39 ± 0.05 ^d^

Different lowercase letters in each vertical column among the same sample group (the LPE group and LSE group) denote statistically significant differences (*p* < 0.05).

**Table 5:** Effects of LPE and LSE on the iodine binding of wheat starch.

Sample		Iodine Binding		
		λ_max_ (nm)	A_635_	Iodine Binding Capacity (A_635_/A_520_)
LPE	0%	626 ^a^	0.52 ± 0.02 ^a^	2.01 ± 0.03 ^a^
	5%	620 ^b^	0.45 ± 0.01 ^b^	1.92 ± 0.01 ^b^
	10%	617 ^bc^	0.36 ± 0.02 ^c^	1.78 ± 0.01 ^c^
	15%	616 ^bc^	0.26 ± 0.01 ^d^	1.62 ± 0.01 ^d^
	20%	614 ^c^	0.25 ± 0.01 ^d^	1.58 ± 0.02 ^d^
LSE	0%	626 ^a^	0.52 ± 0.02 ^a^	2.01 ± 0.03 ^a^
	5%	617 ^b^	0.40 ± 0.02 ^b^	1.75 ± 0.01 ^b^
	10%	616 ^bc^	0.33 ± 0.01 ^c^	1.67 ± 0.01 ^bc^
	15%	614 ^bc^	0.23 ± 0.01 ^d^	1.57 ± 0.02 ^c^
	20%	607 ^c^	0.19 ± 0.01 ^d^	1.25 ± 0.02 ^d^

Different lowercase letters in each vertical column among the same sample group (the LPE group and LSE group) denote statistically significant differences (*p* < 0.05).

## Data Availability

The original contributions presented in the study are included in the article, further inquiries can be directed to the corresponding author.
